# Study protocol for evaluating the current status and needs assessment of health-related characteristics among students at Albert-Ludwigs-University Freiburg

**DOI:** 10.1371/journal.pone.0295166

**Published:** 2023-12-06

**Authors:** Daniel König, Patrick Jendricke, Katharina Poggel, Lena Staab, Albert Gollhofer

**Affiliations:** 1 Department of Sport and Sport Science, University of Freiburg, Freiburg, Germany; 2 Centre for Sports Science and University Sports, Institute for Nutrition, Exercise and Health, University of Vienna, Vienna, Austria; 3 Department for Nutritional Science, Institute for Nutrition, Exercise and Health, University of Vienna, Vienna, Austria; Xiamen University - Malaysia Campus: Xiamen University - Malaysia, MALAYSIA

## Abstract

Today, university students face study conditions that increase the risk of sedentary behavior, unhealthy eating, and the likelihood of stress, anxiety, and depression. Although the situation has likely worsened in recent months due to the COVID-19 pandemic, even in the pre-Corona era, several investigations have demonstrated that the students’ health behaviors could increase the incidence of metabolic risk factors and non-communicable diseases, however, further and detailed information is needed to develop tailored counter-strategies. Therefore, in the present survey, the current health status of students at the Albert-Ludwigs-Universität Freiburg (ALU) will be recorded by various questionnaires. In addition, barriers that could potentially influence their health behavior will be identified, and information on the use and awareness of existing health services will be obtained in order to derive concrete needs for further health promotion activities. The study is designed as a monocentric and prospective study at the ALU; the survey of students’ situation and needs assessments will be conducted in the areas of nutrition, exercise, relaxation and stress reduction, self-management, psychosocial health and socio-demographic correlations via an anonymous and self-administered online questionnaire. Subsequently in two-year intervals, the survey will be repeated. Data will be collected over a period of 4 years. One goal of this survey is to gain more insight into the health situation of university students; another goal is to use the knowledge gained to integrate holistic health measures into the university landscape in a structured and sustainable manner. These health measures will be implemented by using the newly developed student health care management at the ALU (www.sgm.uni-freiburg.de). Every two years, after evaluation of the surveys, the effect of the health measures can be checked and adjusted.

**Trial registration:** ETK: 20–1082; DRKS-ID: DRKS00024088.

## 1. Introduction

In recent years, curricula at many universities in Europe have changed on several levels [1]. Above all, there is a development towards a more rigid curriculum with greater time pressure [2].

Along with the COVID-19 pandemic in spring 2020, face to face instructions ended and teaching and learning had to shift to digital and online formats [3]. In addition, hygiene and social contact regulations have affected the students’ social and academic life [4]. University students are thus increasingly exposed to conditions that resemble the modern world of work with its enhanced workload and exposure to stress [5]. Research over the past several decades in the areas of physical activity [6], nutrition [7], relaxation and stress reduction [8], self-management [9] as well as psychosocial behavior [10] of students has demonstrated a clear need for lifestyle and behavioral improvements.

Compared to all other age groups, university students have had the greatest decline in physical activity and the greatest increase in sedentary behavior across the past decade [6, 11]. Only 20–30% of students in Germany meet the World Health Organization (WHO) guidelines for physical activity [12, 13]. In this context, the differences between the individual faculties at Universities are striking [12]. In addition, there is an increasing trend towards sedentary behavior, especially among female students [14, 15].

Alongside a physically active lifestyle, a healthy and balanced diet contributes significantly to the prevention of chronic diseases such as type 2 diabetes and cardiovascular diseases [7, 16]. In contrast, an unhealthy diet promotes the risk for non-communicable diseases (NCD) and increases NCD health related risk factors even at younger ages [17]. If one compares the food intake in Germany with the recommendations of the German Society for Nutrition [DGE], one finds an excessive consumption of foods of animal origin. In contrast, the consumption of vegetables corresponds to only about one third of the recommendations [18]. Students, in particular, rarely meet recommendations for fruit and vegetable consumption and also deviate unfavorably from recommendations in other food categories [19, 20].

Beginning a university education is accompanied by major changes in life circumstances, often involving significant challenges and changes in students’ lives, such as moving out of the parental home, living alone, identity crisis or an altered daily routine [21]. In Germany, 53% of students feel exposed to a high stress level and 42% to a medium stress level. According to Herbst et al. (2016), female students are more stressed than male students and the type of university or the study program also has an impact on stress experience [22]. Psychological stress often implies reduced mental health and leads to lower performance [23, 24].

Due to the heavy workload with too many courses, exams, and study hours, approximately 50% of students state that their resources and subjective stress limits are exceeded [25]. Good self-management has been shown to have a significant positive impact on greater work and life satisfaction [26] as well as academic performance [27]. Interestingly, female students show higher self-management skills compared to male students. This shows that a differentiated view with separation into genders, study programs or university faculties is necessary to gain a better understanding of the required countermeasures [26].

Over the last few years, alcohol and drug use among students has increased significantly [28]. Students have a remarkably higher alcohol consumption compared to the general population and are more prone to abusive consumption patterns [29]. Male students in particular have more problematic usage patterns compared to female students [30, 31]. Abuse of illicit drugs is also particularly prevalent among young people [32]. Accordingly, students are considered a risk group for problematic consumption behavior, which can lead to various problems at the physical, social, and psychological level [33].

Among students, mental disorders are relatively common. Based on the World Mental Health Survey, Auerbach et al. (2016) reported that 20% of students worldwide have had some form of mental or anxiety disorder or depression in the past 12 months [10]. The prevalence of anxiety disorders peaks globally at age 20–24 years and varies widely by gender and international regions [34]. The reported prevalence of depression among college students varies from 10% to 85%. However, the weighted prevalence of 30.6% is significantly higher than for the general population [35]. The wide range of results shows that we need more diverse research on this question.

The current health situation of students at ALU Freiburg has not yet been surveyed in any study. The aim of the information obtained is to structure holistic health-promoting measures and integrate them into the university landscape in a structured and sustainable manner.

## 2. Project objectives

The primary objective of the situation and needs analysis is the collection of data on health behavior patterns with regard to physical activity, nutrition, relaxation and stress reduction, self-management, psychosocial health as well as life circumstances and the overall relationship with sociodemographic data of students at the ALU Freiburg. The results will be compared with student surveys from other universities but more importantly, the data will be aligned with national and international guidelines to determine if or in how far they are met by the students. As a secondary goal, barriers that influence health will be identified, and information will be obtained on the use and awareness of preexisting health services at ALU or externally. The need for health interventions is derived from both primary and secondary goals of this survey (cf. below).

From the knowledge gained, health measures will be structured and sustainably integrated into the university landscape with the aim of aligning the university with health promotion and disease prevention of its students. These goals will be realized in the respective categories via the following primary and secondary target variables.

### 2.1 Primary target variables

1. Physical activity: The primary outcome measure is the achieved Metabolic Equivalent (MET)-minute count per week in strenuous and moderate activity. This will be compared to WHO guidelines on health-promoting physical activity (600 MET minutes per week). In addition, the average sitting time in hours per day will be quantified. These primary outcome measures will be examined for gender and faculty differences and target groups with below-average physical inactivity and high sedentary behavior identified.

2. Nutrition: The main target variable is the summed value of all 10 identified food categories summarized as the Healthy Eating Index of the National Eating Survey II (HEI-NVS II index). This indicates whether the DGE recommendations of healthy eating are met (≥ 100 points). These primary outcome measures will also–as all following target variables—be examined with respect to gender and faculty differences to identify target groups with a higher-than-average unhealthy eating pattern. Thus, target groups with above-average unhealthy eating behavior can be detected.

3. Relaxation and stress reduction: The primary outcome measure is stress experience as measured by the summed score of the Perceived Stress Scale-10(PSS-10). This will be applied to examine how many students have a moderate to high stress experience (PSS-10 sum > 13). The 5 coping strategies of the Stress and Coping Inventory (SCI) represent another outcome measure of the primary objective. They are each assigned to a below-average or above-average performance level in order to determine target groups with above-average stress experience.

4. Self-management: The main target variable is the self-management competence of the students, measured by the sum value of the SMST. It examines the number of students who have mediocre to very poor self-management skills (SMST sum score < 13). As such, the identification of target groups with below-average self-management skills is made possible.

5. Psychosocial health, consisting of a) alcohol and drug use, b) depression, c) anxiety disorders, and d) sleep disorders:

(a) Alcohol consumption, as assessed by the AUDIT sum score, will be the primary outcome. The extent to which students exhibit problem drinking patterns, symptoms of alcohol dependence and characteristics of risky consumption (AUDIT sum > 11) will be addressed.
(b) Harmful drug use, quantified by the sum score of the DUDIT (men: ≥ 6; women: ≥ 2), is another outcome variable of the primary target.
(c) In addition, the prevalence of depression will be surveyed (PHQ-9: cut-off ≥ 10). The severity of a possible depression will be gauged by a dimensional assessment of the questionnaire (10–14 mild depression; 15–19 moderate depression; 20–27 severe depression).
(d) Generalized anxiety disorder, as scored by the GAD-7 (cut-off ≥ 10), is another primary outcome criterion. Specific severity (mild, moderate, severe) of generalized anxiety disorder will be graded by the dimensional score of the GAD-7.
(e) Insomnia represents the final primary outcome measure. Using the ISI(cut-off ≥ 10), the extent to which students suffer from insomnia will be quantified.

6. Covid-19-Infection: The COVID-19 pandemic has had a significant impact on students’ lives across multiple domains. In addition to exploring inquiries regarding their infection status, frequency, and potential symptoms associated with "Long-Covid," we will utilize the COVID-19 Student Stress Questionnaire (CSSQ) to assess the stressors experienced by students throughout and following the pandemic. This questionnaire has been specifically designed and evaluated to assess COVID-19-related sources of stress among university students and is therefore well suited to address related problems [36].

### 2.2 Secondary target variables

Secondary outcome measures are used to identify the most common barriers for students to make infrequent or no use of health-related services, measured using a five-point scale (from "very important" to "not important").

Moreover, the use of existing offerings and their level of awareness will be asked in a four-step response format (used so far; known but not used; not known but interested; not known and not interested).

In a final step, group-specific barriers to the use of existing services as well as their awareness will be identified. These secondary outcome variables are additionally examined for gender and faculty differences.

## 3. Study population

### 3.1 Inclusion and exclusion criteria

Only students from the 11 faculties of the ALU Freiburg are allowed to participate in the needs analysis. For this purpose, the following inclusion and exclusion criteria have been defined:

Inclusion criteria:

- Enrolled at the ALU Freiburg
- ≥18 years of age

Exclusion criteria:

- Institutes or universities that do not belong to ALU Freiburg (private institutes, university of education, universities of other sponsors in Freiburg, etc.).
- Employees of the ALU
- Doctoral students
- Students with physical impairments that prevent them from moderate and/or strenuous physical activity.
- Students with an impairment/illness that strongly influences their dietary behavior
- Students who have a severe psychological impairment

### 3.2 Number of participants in the studies

According to recent student statistics at ALU Freiburg, 24,391 students from over 100 nations are enrolled in 180 study programs at 11 faculties. Of these, 12,908 are female students and 11,483 are male students. The goal is to have > 2% of the students in each faculty participate in the survey to ensure a representative sample of the faculties. Thus, in total > 500 university students should be surveyed [37].

### 3.3 Recruitment measures

To ensure that all university students are actually informed about the survey and can potentially be recruited to participate in the needs assessment, a link to the survey will be provided via the University of Freiburg’s central learning platform ILIAS. Students can use this link to obtain both information and access to the survey. Further recruitment activities are to take place via the social platforms of the Student Health Management (SGM). Likewise, the SGM kick-off will be used to advertise participation in the needs assessment. In addition to the application, direct participation via provided PC access will be made possible.

## 4. Methodology and implementation

### 4.1 Informing and obtaining the consent of the students

Before answering the questionnaire, students will be informed about the scope of the survey. For that purpose, a participant information sheet is provided on the website of the survey. In layman’s terms, this contains the background, the objective and the content framework of the survey. Moreover, the participant information sheet lists and describes detailed information on data management and data protection. As the survey is completely anonymous on a website and there is no prior contact with the examiners, participation in the survey is based on the assumption that the students have given their informed consent. The login process via the University of Freiburg ensures that no minors participate in the surveys.

### 4.2 Questionnaire survey

At the start of the study and subsequently at two-year intervals until 2026, situation and needs analyses will be conducted using validated and evaluated questionnaires. Data will be collected via an anonymous and self-administered online questionnaire via Social Science Survey [SoSci Survey] [38]. All questionnaires applied have been validated and have already been employed in various studies addressing comparable issues. No modifications have been made to the questionnaires in the context of the present investigations that would necessitate a re-validation.

#### 4.2.1 Physical activity

Physical activity will be assessed with the International Physical Activity Questionnaire (IPAQ) [39]. In addition, following Wallace et al. (2000), the weekly sitting time (WST) for studying, at the computer and for screen-related work will be queried via three items [40]. Based on Kramer and Fuchs (2010), possible barriers and barrier management in the process of sports participation will be identified via 13 items [41].

#### 4.2.2 Nutrition

The German Health Examination Survey (DEGS) food frequency questionnaire for national health and nutrition monitoring is used to survey dietary behavior [42]. This consumption frequency questionnaire (FFQ) will be applied to collect the consumption frequency and usual portion amounts of a total of 53 foods consumed in the last four weeks. To assess food choices, dietary patterns will be based on the Healthy Eating Index of the National Eating Survey II (HEI-NVS II) [43]. Here, the 53 food items are divided into food categories, which are compared and evaluated on the basis of the recommendations of the German Nutrition Society (DGE) [44]. In accordance with Hilger et al. (2017), 22 items will be provided to determine which barriers prevent an unhealthy diet [45].

#### 4.2.3 Relaxation and stress reduction

In order to assess the stress experience of university students, the Perceived Stress Scale (PSS) will be applied [46, 47]. The self-efficacy feeling and the feeling of helplessness of the last month are determined on the basis of 10 items. Another component is the assessment of specific coping strategies in response to a situation experienced as stressful using the "Stress and Coping Inventory" (SCI) [48].

#### 5.2.4 Self-management

With the help of the "Self-Management Self-Test" (SMST) the students will be asked how they subjectively assess their self-management competence. The SMST is composed of five items. Five areas of self-management are queried: cognition, relationship, planning, decision-making, and action [49].

#### 4.2.5 Psychosocial health

To assess anxiety disorders, the Generalized Anxiety Disorder Scale (GAD-7) questionnaire will be used, which asks seven items about generalized anxiety disorder as well as the symptom severity of generalized anxiety [50–52]. The Patient Health Questionnaire [PHQ-9] serves as a screening instrument for the assessment of depressiveness. These inquiries about the presence and frequency of nine diagnostic criteria of depression within the previous two weeks [53–55]. Insomnia will be quantified by the German validated Insomnia Severity Index [ISI] [56]. With seven items referring to the last month, the severity of a sleep disorder will be surveyed. By means of the questionnaire 11-item Drug Use Disorders Identification Test (DUDIT), substance use disorders are identified [52, 57, 58]. In addition, self-assessment of alcohol consumption will be evaluated by weekly consumption amount via the 10-question Alcohol Use Disorders Identification Test (AUDIT) questionnaire [52, 59, 60]. Barriers to using health services in the event of future emotional problems will be assessed using six items from the WHO International Student Initiative on Mental Health [61] and three items based on Hoffmann et al. (2008) and Chung et al. (2018) [62, 63].

#### 4.2.6 Sociodemographic data

The sociodemographic data serve as a way to examine the collected data on the respective domains for differences and similarities in terms of sociodemographic characteristics. Following Ebert et al. (2019), the questionnaire asks about the following characteristics [61]: Gender, age, height, weight, intended degree, degree program, faculty affiliation, number of university semesters, physical and/or mental impairment; if any: Type of impairment, relationship status, child or children, migration background, if yes: of which nationality, current living situation [shared apartment, student dormitory, alone, with partner, parental home], financing of studies [monthly money available and monthly rental costs], subjective social status, satisfaction with life and studies, commitment to studies, general state of health (Health-Related Quality of Life Questionnaire [HRQL-4 Core]) [64].

#### 4.2.7 Barriers and utilization of health services

Regarding physical activity, nutrition, relaxation and stress reduction, self-management, and psychosocial health, response formats for both barriers and utilization of health services were designed based on the WHO International Student Initiative by Ebert et al. (2019) [61]. Three other possible ones are based on Hoffmann et al. (2017) and Chung et al. (2018) [62, 63]. Possible reasons against use are queried with a five-step response format [1–5], which differentiates the strength of hindrance of the situation from "very important" to "important" and "moderately important" to "not very important" and "not important". The utilization and awareness of already existing offers are operationalized by a four-stage response format [1–4], which subdivides the utilization strength of the offers from "used so far" to "known but not used" and "not known but would have interest" to "not known and no interest". In addition, there will be an option of expressing individual wishes regarding the selection of offers in a free text field.

## 5. Biometry

The present study is an exploratory study conducted monocentrically and prospectively at ALU Freiburg in Germany. Based on the 2014 German Student Survey on sample selection and representativeness of data, a sample of > 500 students is recommended to obtain a sufficiently large number of students in the different subject distributions [37]. Approximately 0.9% of all students at the universities took part in a survey in the winter semester 2012/13. Participation varied between the universities by 0.6–1.6%. It was pointed out that for representative statements, it is not so much the relative proportion of the sample to the population that is important, but the absolute size of the sample. With this in mind, > 500 students are expected to participate in the survey in the present investigation, thus creating a representative sample.

Data analysis will be conducted using IBM SPSS Statistics version 26.0. All tests are two-sided and the significance level will be set at α = 5%. Effect size d will be calculated according to Cohen (1988) and interpreted as follows: < 0.2 (no effect); ≥ 0.2 (small effect); ≥ 0.5 (moderate effect); and ≥ 0.8 (large effect) [65]. To test the data for normal distribution, if necessary, the Shapiro-Wilk test will be chosen due to the large sample size.

In principle, the data collected on prevalence in relation to exercise, nutrition, relaxation and stress reduction, self-management, psychosocial health, and sociodemographic data of students of the ALU Freiburg will be illustrated and presented by means of descriptive statistics and graphs, since this is primarily a description of the opportunity sample. Therefore, the number of the sample population is given as N and as a percentage. For interval-scaled data, the mean (MW) and ± standard deviation (SD) will also reported. Along with the MW and SD, the median will also be given for the descriptive analysis of the parameters.

In order to investigate whether the national and international guidelines are met by students in the respective areas, a Chi-square test will be performed by a cross-tabulation. At the same time, the cross-tabulations will also be used to compare the respective faculties. Using a t-test for independent samples, differences in characteristic expressions, such as gender and faculty differences, will be analyzed. If there is no normal distribution, the Mann-Whitney U-test will be applied.

In addition to highlighting differences, the sociodemographic variables will be examined together with other independent variables from the respective categories in a multiple regression. Spearman-Rho correlation or person correlation in the case of normal distribution will be used to check whether there are correlations between the study parameters.

**Fig 1:**
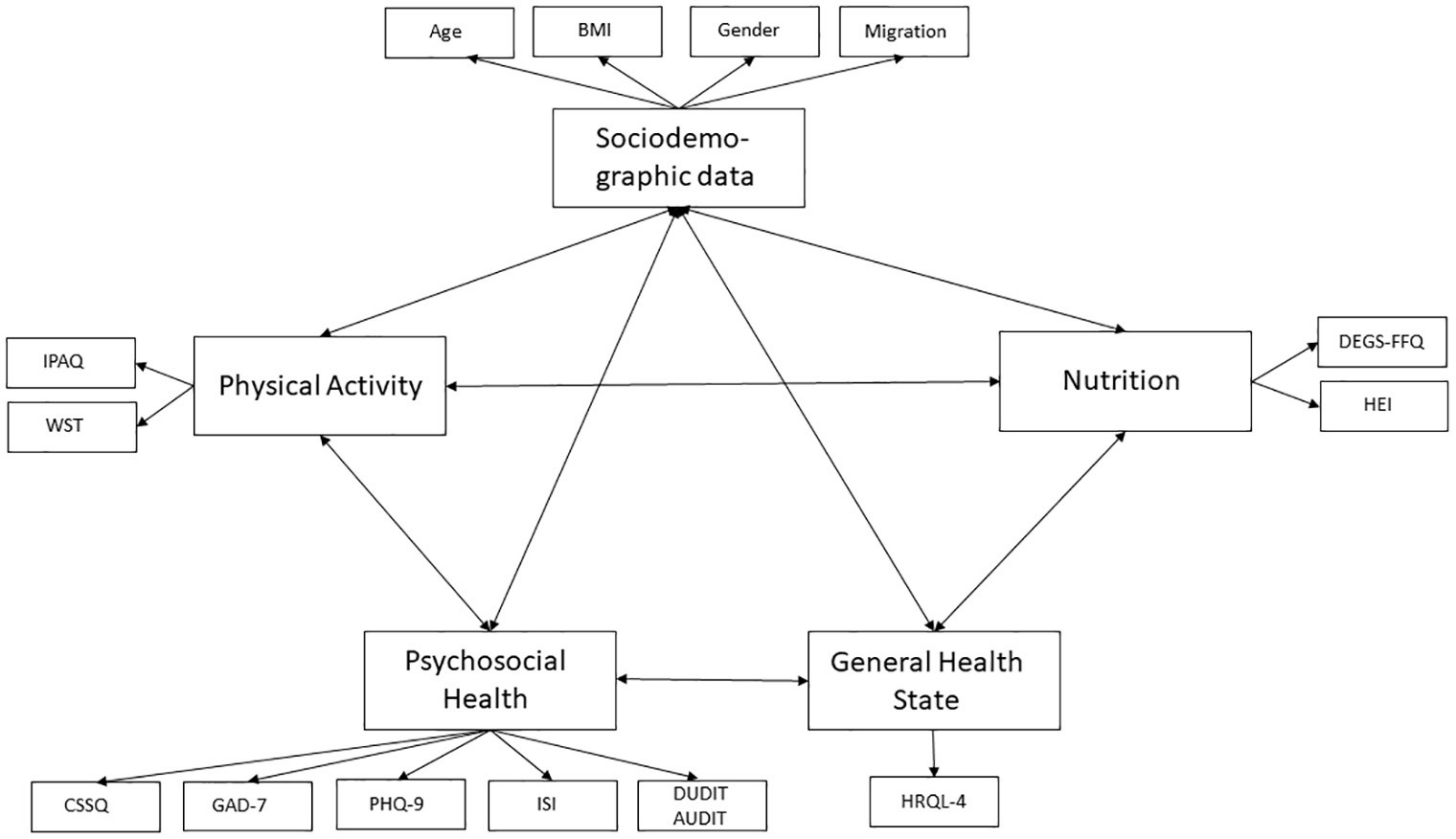
Structural equation modelling (SEM) path diagram of potential associations between the latent variables and their corresponding observed variables. Sociodemographic data: Age, BMI, Gender, Migration background. Nutrition; DEGS-FFQ: German Health Examination Survey; HEI: Healthy Eating Index of the National Eating Survey II. General Health State: HRQL-4: Health related quality of life. Psychosocial Health: GAD-7: Generalized Anxiety Disorder Scale; PHQ-9: Patient Health Questionnaire; ISI: German validated Insomnia Severity Index; DUDIT: Drug Use Disorders Identification Test; AUDIT: Alcohol Use Disorders Identification Test; COVID-19 Student Stress Questionnaire (CSSQ). Physical Activity: IPAQ: International Physical Activity Questionnaire; WST: weekly sitting time.

Furthermore, to understand if there are causal relationships between the variables investigated, “Structural Equation Modeling” and the creation of causal models will be performed. Fig 1 shows the path diagram representing the theoretical associations between the latent and observed variables.

## 6. Data management and data protection

Data management will be carried out in accordance with Good Clinical Practice (GCP) requirements and the provisions of the European Union Data Protection Regulation (EU-DSGVO). The data for the study evaluation will be collected per student via an online questionnaire using SoSci Survey. Large-scale scientific survey projects using SoSci Survey have been successfully conducted for the realization of such studies for many years. After data collection, SoSci Survey exports the data correctly, completely and immediately in the form of a spreadsheet to a password-protected computer and automatically deletes it irrevocably from the SoSci Survey server after 3 months. No one except the study director and the participating researchers (page 1) are allowed to view the password-protected and encrypted spreadsheet.

All student-relevant study data will be stored anonymously. Name, private address, personalized e-mail address, matriculation number or other identification numbers of students will not be requested. Merely a random number will be assigned to each student. Only after the students have voluntarily sent their number to the scientists involved (e-mail: sgm@sport.uni-freiburg.de), will it be possible to assign the person at great expense in terms of time, cost and manpower.

## 7. Discussion

Previous research has shown that university students are increasingly likely to have unhealthy lifestyles and are exposed to growing stress in their studies as well as in their daily lives. Particularly in the areas of physical activity [6], nutrition [7], stress [8] and self-management [9] as well as psychosocial behavior of students [10], it has been clearly shown that there is a need for lifestyle and behavioral improvement. At the moment, this situation is further exacerbated by the current COVID-19 pandemic [66]. The health situation of students at the ALU Freiburg has not yet been surveyed in any study, which reveals the need for an up-to-date situation and needs analysis.

The primary objective of this survey is the collection of data on health behavior patterns with regard to the above-mentioned items as well as an assessment of life circumstances and the overall association of these findings with sociodemographic data of students at the ALU Freiburg. Therefore, the situation and needs analyses will be carried out at the beginning of the project and subsequently every two-years.

Based on the results, the aim of ALU Freiburg is to establish a university-wide network in the field of student health promotion and thus to establish a sustainable student health management (SGM– www.sgm.uni-freiburg.de). In the long term, the aim is to create an environment for students that promotes both, health and good performance in their studies.

Through the repetition of surveys every two years, the health situation of the students will be repeatedly recorded in order to subsequently develop new health offers dependent on the results of the actual needs assessment. Consequently, this measure makes up an important part of the overall Student Health Management (SGM) project.

In conformity with the current body of literature on student health, it is equally apparent that in some outcome variables, there is a strong variation regarding gender as well as between faculties [12]. Thus, the study aims to collect as diverse information as possible on the age, gender, and program affiliation of students in order to create tailored programs for different student groups.
